# Academic stress profiles and bedtime procrastination among college students: the mediating role of rumination and the moderating effect of perceived stress

**DOI:** 10.3389/fpsyg.2026.1784775

**Published:** 2026-04-22

**Authors:** Bingchi Xiang, Guocang Li, Wang Xiang

**Affiliations:** 1 Research Center for Artificial Intelligence and Future Education, Wenzhou University, Wenzhou, China; 2 School of Management, Henan University of Technology, High-Tech Zone, Zhengzhou, China; 3 Cheung Kong School of Journalism and Communication, Shantou University, Shantou, China

**Keywords:** academic stress, bedtime procrastination, latent profile analysis, perceived stress, rumination

## Abstract

**Background:**

While academic stress is a recognized predictor of sleep disturbances, traditional variable-centered research often obscures the population heterogeneity within this relationship. Adopting a person-centered perspective, this study identified latent configurations of academic stress among college students and examined the psychological mechanisms linking these profiles to bedtime procrastination (BPS).

**Methods:**

A sample of 741 Chinese university students completed validated measures of academic stress, rumination dimensions, perceived stress, and BPS. Latent Profile Analysis (LPA) was utilized to classify academic stress subtypes. Subsequently, a moderated mediation model was tested to evaluate the parallel mediating roles of two rumination dimensions—symptom rumination and reflection—and the moderating effect of perceived stress.

**Results:**

LPA identified four distinct latent profiles: Pressure-Insensitive (11%), Well-Adapted (32%), Commonly Burdened (45%), and Overwhelmed (12%). The *Overwhelmed* group exhibited the highest BPS scores (*M* = 0.325), significantly higher than the *Pressure-Insensitive* group (*M* = −0.283). Mediation analysis revealed divergent cognitive pathways: Symptom rumination functioned as a maladaptive mediator that increased BPS (β = 0.24, *p* = 0.002), whereas reflection acted as an adaptive mediator that buffered the adverse effects (β = −0.22, *p* < 0.01). Notably, the *Overwhelmed* profile was the strongest predictor of symptom rumination (β = 1.57, *p* < 0.001). Furthermore, perceived stress significantly moderated the link between the *Commonly Burdened* profile and symptom rumination (Interaction B = 0.27, *p* = 0.009). Simple slope analysis confirmed that this maladaptive pathway was activated only under high levels of perceived stress (Slope = 0.39, *p* < 0.01) but remained insignificant at low levels.

**Conclusion:**

These findings underscore the dual nature of rumination and the “gating” role of subjective appraisal in the stress-sleep relationship. The results suggest that stratified interventions targeting specific cognitive-emotional deficits within different stress profiles are essential for mitigating sleep self-regulation failures among university studencvts.

## 1 Introduction

### 1.1 Research background and problem statement

Sleep deficiency has evolved into a pervasive public health crisis among university populations globally. It precipitates severe consequences for cognitive functioning and psychological wellbeing (Medic et al., 2017). Recent review-level evidence further highlights the widespread nature of sleep-related problems in university populations. A systematic review and evidence map reported that higher education students slept an average of 6.95 h on weekdays and that the overall proportion of sleep problems was approximately 48% across the included studies (Bjørnnes et al., 2021). Moreover, a recent systematic review and meta-analysis found that the pooled prevalence of insomnia symptoms among undergraduate university students was 46.9% (95% CI = 40.1–53.6%) (Spyridonidis et al., 2025). While earlier research primarily focused on sleep disorders such as insomnia, contemporary scholarship has increasingly directed attention toward bedtime procrastination. Kroese and colleagues defined bedtime procrastination as the failure to go to bed at the intended time despite having no external circumstances that prevent one from doing so (Kroese et al., 2014). Unlike insomnia, which is generally characterized by difficulty initiating or maintaining sleep, bedtime procrastination represents a volitional failure in self-regulation involving the delay of sleep onset (Kroese et al., 2016). Empirical evidence indicates that bedtime procrastination is robustly associated with sleep deprivation, daytime fatigue, and reduced self-control capacity (Exelmans and Van den Bulck, 2021a; Sirois et al., 2019). Recent review-level evidence has further strengthened the importance of this construct in university populations. Specifically, a recent systematic review and meta-analysis reported that bedtime procrastination was significantly associated with depression, anxiety, and perceived stress among university students, with stress showing the strongest association (Azeem et al., 2026). Moreover, a recent meta-analytic reliability generalization study demonstrated that the Bedtime Procrastination Scale yields generally acceptable to high reliability across studies. Nevertheless, while these studies consolidate the correlates and psychometric basis of bedtime procrastination, they remain primarily variable-centered and provide limited insight into whether distinct patterns of academic stress confer differential risk for bedtime procrastination through specific cognitive mechanisms (Oyar et al., 2026). Against this background, academic stress may represent a particularly important antecedent of bedtime procrastination among college students.

For college students, the transition to higher education entails navigating an environment characterized by intense academic competition and anxiety regarding future career prospects. Consequently, academic stress has been identified as a predominant antecedent of poor sleep hygiene (Lund et al., 2010). Studies have consistently demonstrated that high levels of study-related stress are correlated with shorter sleep duration and lower sleep quality (Ahrberg et al., 2012; Deng et al., 2021). However, the existing literature investigating the link between academic stress and bedtime procrastination relies heavily on variable-centered approaches. These methods typically utilize aggregate scores to represent stress levels and assume population homogeneity (Laursen and Hoff, 2006). This methodological limitation obscures the possibility that students may experience distinct configurations of stressors. For instance, some individuals may face high parental expectations while maintaining low peer competition, whereas others may experience comprehensive pressure across all domains. Latent Profile Analysis (LPA) offers a person-centered alternative capable of identifying unobserved subgroups that share similar stress patterns (Wang and Hanges, 2011). Ignoring this heterogeneity may lead to an oversimplified understanding of how specific academic burdens differentially predict sleep outcomes. Previous research has begun to apply latent profile analysis to stress in university students. For instance, Kökçam et al. identified distinct stress profiles and demonstrated that these profiles differed meaningfully in psychological resilience and emotional intelligence (Kökçam et al., 2022). However, existing person-centered studies have paid limited attention to academic stress specifically, and have not sufficiently examined how different academic stress profiles may be linked to bedtime procrastination through differentiated cognitive and perceptual mechanisms.

### 1.2 Theoretical framework: conservation of resources theory

To explicate the mechanism through which academic stress translates into BPS, the Conservation of Resources (COR) Theory provides a robust theoretical lens (Hobfoll, 1989). Hobfoll postulated that individuals strive to acquire, maintain, and foster resources. Stress occurs when these resources are threatened or lost (Tian and Deng, 2007). According to COR theory, students investing substantial cognitive and emotional resources to cope with academic demands during the day experience a state of ego depletion by nightfall. In this context, BPS emerges as a compensatory mechanism. Students engage in recreational activities late at night to regain a sense of autonomy and recover lost resources, a phenomenon often described as revenge bedtime procrastination (Nauts et al., 2019).

### 1.3 The mediating role of rumination dimensions

A critical psychological process that exacerbates resource depletion is Rumination. Defined as repetitive and passive thinking about distress and its causes, rumination sustains physiological and cognitive arousal that is incompatible with sleep initiation (Guastella and Moulds, 2007). High levels of stress are known to trigger ruminative thinking, which subsequently consumes the limited self-control resources required to adhere to a bedtime routine (Pillai et al., 2014).

However, the Response Styles Theory proposed by Nolen-Hoeksema suggests that rumination is not a monolithic construct (Nolen-Hoeksema, 1991). It comprises distinct dimensions that may function differently (Treynor et al., 2003). Treynor and colleagues further differentiated rumination into brooding and reflection. In addition, previous psychometric research has suggested that symptom rumination represents a symptom-focused component of rumination, capturing repetitive attention to one’s emotional and somatic symptoms of distress and their possible consequences (Burwell and Shirk, 2007). Brooding involves a passive comparison of one’s current situation with unachieved standards and is strongly linked to depression (Joormann et al., 2006). In contrast, reflection involves an active and purposeful turning inward to engage in cognitive problem-solving (Takano and Tanno, 2009). While brooding is generally maladaptive, reflection can be adaptive as it may facilitate the processing of negative emotions (Watkins, 2008). Although symptom rumination and brooding are both maladaptive forms of repetitive thinking, symptom rumination places greater emphasis on persistent fixation on distress symptoms themselves, whereas brooding emphasizes negative self-evaluative comparison between one’s current state and desired standards (Nolen-Hoeksema, 1991). Reflection differs from both in that it is more purposeful and problem-oriented, and may therefore contribute to emotional processing rather than merely prolonging distress (Nolen-Hoeksema et al., 2008).

Prior research on BPS has frequently treated rumination as a unitary negative variable. This approach neglects the potential buffering effect of adaptive reflection. It is plausible that while stress-induced symptom rumination and brooding exacerbate procrastination by heightening pre-sleep arousal and emotional fixation, stress-induced reflection might facilitate problem resolution and reduce the anxiety that drives sleep delay (Roelofs et al., 2006; Treynor et al., 2003). Therefore, this study aims to disentangle these effects by examining the parallel mediating roles of symptom rumination, brooding, and reflection.

### 1.4 The moderating role of perceived stress

While objective academic profiles provide a structural view of stress, they do not fully account for individual differences in appraisal. The Transactional Model of Stress and Coping emphasizes that the impact of a stressor depends fundamentally on the individual’s subjective appraisal (Ma et al., 2021). Perceived Stress, which reflects the degree to which situations in one’s life are appraised as stressful, uncontrollable, or overloading, acts as a critical boundary condition (Han and Yang, 2009).

Two students belonging to the same “moderate stress” profile may exhibit vastly different psychological outcomes depending on their subjective perception. For individuals with high perceived stress, the objective academic burden is more likely to activate maladaptive cognitive patterns such as symptom rumination (Hammen, 2005). Conversely, those with low perceived stress may maintain psychological equilibrium despite the objective load. Understanding the interaction between objective stress profiles and subjective perceived stress is essential for identifying risk-susceptible populations who are most vulnerable to the resource-depleting effects of academic stress.

### 1.5 The present study and hypotheses

In light of these gaps, the present study adopts a combined person-centered and variable-centered approach to construct a moderated mediation model. First, we employ Latent Profile Analysis to identify distinct subtypes of academic stress among college students. Second, we examine whether these profiles predict BPS through the differentiated mediating pathways of rumination dimensions. Finally, we investigate whether perceived stress moderates the link between stress profiles and rumination.

Based on the theoretical framework and empirical evidence, we propose the following hypotheses:

*H1:* There exists significant heterogeneity in academic stress among college students, which can be classified into distinct latent profiles.

*H2*: Academic stress profiles characterized by higher burdens significantly predict higher levels of bedtime procrastination.

*H3:* The dimensions of rumination play parallel but distinct mediating roles. Specifically, symptom rumination and brooding will positively mediate the relationship (exacerbating BPS), whereas reflection will negatively mediate the relationship (buffering BPS).

*H4:* Perceived stress moderates the first stage of the mediation process, acting as a risk activator that strengthens the effect of stress profiles on maladaptive rumination.

**FIGURE 1:**
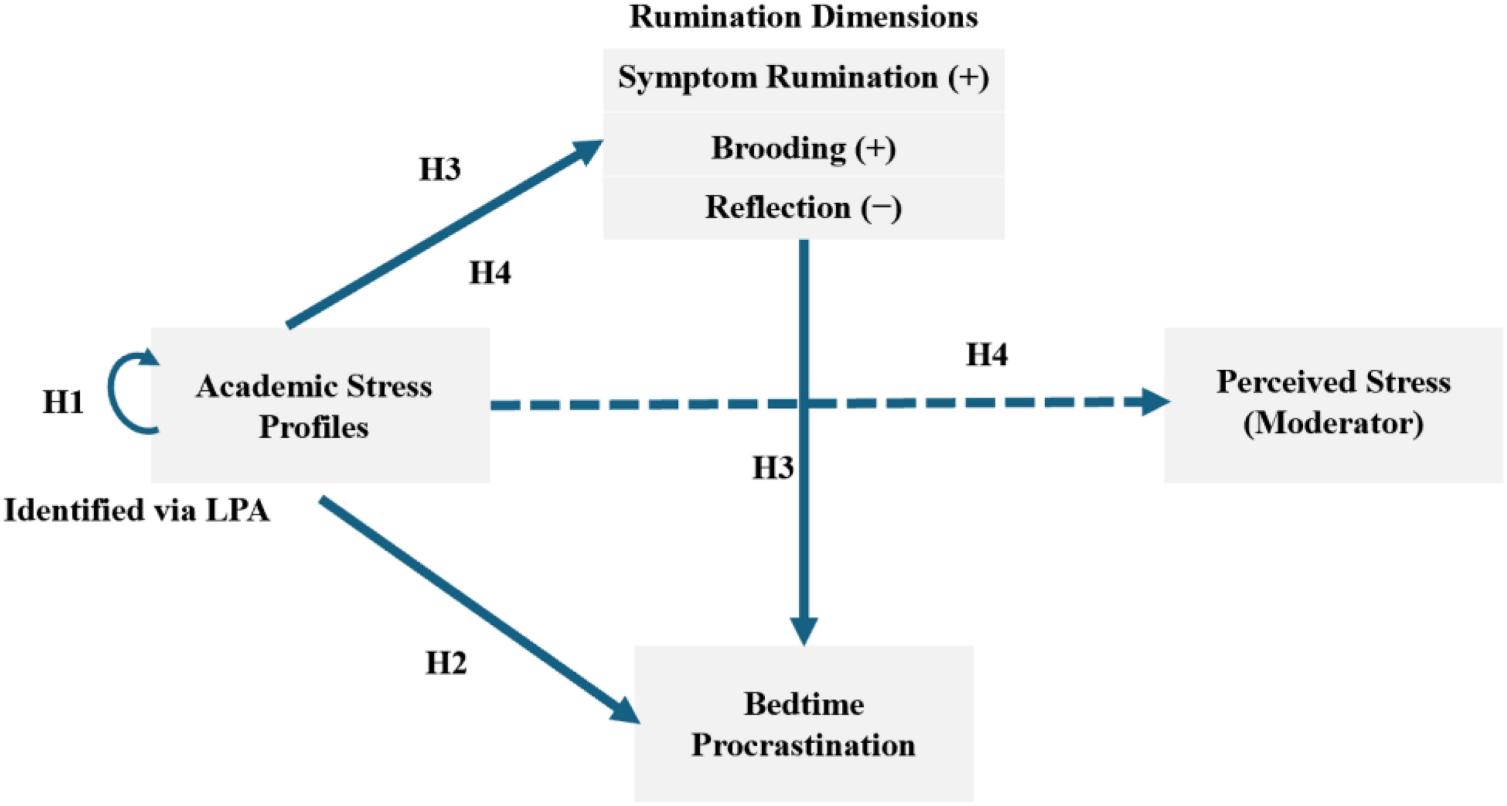
Research hypothesis model.

Figure 1 illustrates the conceptual framework and the hypothesized relationships among the study variables. The model establishes a moderated mediation framework to systematically explicate the impact of academic stress on bedtime procrastination. Specifically, the latent profiles of academic stress identified via Latent Profile Analysis are positioned as the independent variable on the left side of the figure (corresponding to Hypothesis 1). The dependent variable, bedtime procrastination, is positioned on the right.

Central to the framework is the mediation mechanism comprising the three dimensions of rumination: symptom rumination, brooding, and reflection. The model proposes that academic stress profiles influence bedtime procrastination indirectly through these parallel pathways (corresponding to Hypothesis 3) while also retaining a direct predictive path (corresponding to Hypothesis 2). Furthermore, perceived stress is integrated as a moderator. The model posits that perceived stress influences the strength of the association between academic stress profiles and the rumination dimensions (corresponding to Hypothesis 4). This structure constitutes a comprehensive moderated mediation model.

## 2 Materials and methods

### 2.1 Participants and procedure

This study employed a convenience sampling strategy to recruit university students through an online survey platform (Wenjuanxing). The data collection period spanned from December 24, 2025, to December 30, 2025. Prior to accessing the questionnaire, all participants were presented with an informed consent form detailing the anonymity of the study, the voluntary nature of participation, and their right to withdraw at any time. To reduce potential common method bias, participants were further informed that there were no right or wrong answers and were encouraged to respond honestly based on their actual experiences. In addition, all measures were administered using standardized instructions and well-established scales.

No formal a priori sample size calculation was conducted before data collection. Instead, the sample size was determined based on recruitment feasibility and the aim of obtaining a sufficiently large sample for the planned analyses. Given that the present study involved latent profile analysis and moderated mediation analysis, we attempted to recruit as many eligible participants as possible to ensure adequate statistical stability.

A total of 768 initial responses were collected. To ensure data quality, rigorous data cleaning procedures were applied based on the following exclusion criteria: (1) refusal to provide informed consent; and (2) response irregularities, such as duplicate submissions or completing the survey in an unrealistically short time. Specifically, 5 participants were excluded for declining consent, and 18 responses were removed due to redundancy or validity checks. Consequently, a final valid sample of 741 participants was retained for analysis, yielding an effective response rate of 97.12%.

The demographic composition of the final sample was diverse. In terms of gender distribution, the sample consisted of 309 males and 432 females. The mean age of the participants was 19.46 years, with a standard deviation of 1.49. Regarding academic standing, the distribution covered all undergraduate levels: 435 freshmen (58.7%), 105 sophomores (14.2%), 132 juniors (17.8%), 54 seniors (7.3%), and 15 students in their fifth year or above (2%). Furthermore, the participants represented a wide range of disciplinary backgrounds. Students from Science, Technology, Engineering, and Agriculture (STEM) majors constituted the largest group (47%), followed by Humanities and Social Sciences (27%), Medicine and other disciplines (18.3%), and Arts and Sports (7.7%).

### 2.2 Measures

#### 2.2.1 Academic stress

Academic stress was assessed using the Chinese version of the multidimensional academic stress scale (Tian and Deng, 2007). This instrument comprises eight sub-dimensions: prospect stress, goal stress, task demand stress, competition stress, frustration stress, parental stress, expectation stress, and environmental stress. Participants rated items on a Likert scale, with higher scores indicating greater perceived academic burden. In the present study, the Cronbach’s alpha coefficient for the total scale was 0.95, and a McDonald’s omega coefficient of 0.96.

#### 2.2.2 Bedtime procrastination scale

Bedtime procrastination was measured using the Chinese version of the Bedtime Procrastination Scale developed by MX (Ma et al., 2021). This scale evaluates the extent to which individuals voluntarily delay going to bed without external reasons. Items were scored on a Likert scale ranging from 1 (almost never) to 5 (almost always). The Cronbach’s alpha for this scale in the current study was 0.89, and a McDonald’s omega coefficient of 0.90.

#### 2.2.3 Rumination

Rumination was assessed using the Chinese version of the Ruminative Responses Scale (RRS) (Han and Yang, 2009). Consistent with Treynor’s factor structure, we focused on three dimensions: symptom rumination, brooding, and reflection. Higher scores represent a higher tendency to engage in ruminative thinking. The internal consistency for the subscales ranged from 0.83 to 0.91 and McDonald’s omega coefficients ranging from 0.88 to 0.89. Specifically, the McDonald’s omega coefficients were 0.89 for symptom rumination, 0.88 for brooding, and 0.89 for reflection.

#### 2.2.4 Perceived stress

Subjective perceived stress was measured using the Chinese version of the Perceived Stress Scale (PSS-10) (Lu et al., 2017). This tool assesses the degree to which situations in one’s life are appraised as stressful. Higher scores indicate higher levels of perceived stress. In the present study, the scale demonstrated good internal consistency, with a Cronbach’s alpha coefficient of 0.87 and a McDonald’s omega coefficient of 0.88.

#### 2.2.5 Control variables

Based on prior literature suggesting that sleep behaviors and sleep quality in young adults and university students may vary according to demographic and health-related characteristics, including sex, age, self-rated health, and technology use (Fatima et al., 2016; Leow et al., 2023; Schmickler et al., 2023), this study controlled for gender, age, grade level, physical health status, and daily duration of mobile phone use for entertainment.

### 2.3 Statistical analysis

Data management and statistical analyses were conducted using SPSS 26.0 and Mplus 8.3 software. The analysis proceeded in four stages. Before the main analyses, the normality of the core continuous variables was examined using skewness and kurtosis indices. First, descriptive statistics and Pearson correlation analysis were performed to examine the relationships among core variables. Second, Latent Profile Analysis (LPA) was conducted using Mplus 8.3 to identify distinct subtypes of academic stress. Model fit was evaluated using indices including AIC, BIC, aBIC, Entropy, LMRT, and BLRT. Third, the differences in variables across the identified profiles were tested using ANOVA and the BCH method. Finally, the PROCESS macro (Model 4 and Model 59) was utilized to test the mediation effects of rumination and the moderated mediation effect of perceived stress. The significance of regression coefficients was verified using the bias-corrected percentile Bootstrap method with 5,000 resamples. Given the use of latent profile analysis and moderated mediation analysis, the final sample size was considered adequate to support stable estimation of the proposed models. In addition to Cronbach’s alpha, McDonald’s omega coefficients were calculated to provide a more comprehensive assessment of the internal consistency of the scales used in the present study.

## 3 Results

The skewness and kurtosis values of the core continuous variables were within acceptable ranges, suggesting that the data were approximately normally distributed. Before conducting the main analyses, the reliability and distributional properties of the study variables were examined. The Cronbach’s alpha and McDonald’s omega coefficients indicated satisfactory to excellent internal consistency for all scales and subscales. In addition, the skewness and kurtosis values of the core continuous variables fell within acceptable ranges, suggesting that the data were approximately normally distributed and that subsequent parametric analyses were appropriate.

### 3.1 Descriptive statistics and correlation analysis

Pearson correlation analysis was employed to examine the interrelationships among the core variables and their dimensions, with detailed statistical results presented in Table 1. Overall, the analysis revealed widespread and significant positive correlations among academic stress, bedtime procrastination behavior, rumination, and perceived stress. These findings establish the necessary empirical premise for the subsequent latent profile analysis.

**TABLE 1:** Correlation analysis results.

Variable	1	2	3	4	5	6	7	8	9	10	11	12	13	14
1- Academic stress	1	1	1	1	1	1	1	1	1	1	1	1	1	1
2- Future pressure	0.859***													
3- Goal pressure	0.918***	0.784***												
4- Task demand pressure	0.937***	0.814***	0.823***											
5- Competitive pressure	0.928***	0.780***	0.848***	0.859***										
6- Frustration pressure	0.859***	0.594***	0.737***	0.766***	0.776***									
7- Parental pressure	0.842***	0.630***	0.706***	0.728***	0.751***	0.765***								
8- Expectation pressure	0.856***	0.636***	0.812***	0.771***	0.767***	0.766***	0.722***							
9- Environmental pressure	0.816***	0.602***	0.680***	0.754***	0.718***	0.788***	0.671***	0.709***						
10- Sleep procrastination	0.215***	0.294***	0.194***	0.217***	0.216***	0.112**	0.135***	0.148***	0.092*					
11- Ruminative thinking	0.494***	0.501***	0.451***	0.460***	0.467***	0.349***	0.421***	0.359***	0.400***	0.297***				
12- Compulsive meditation	0.498***	0.526***	0.475***	0.467***	0.461***	0.347***	0.409***	0.357***	0.350***	0.275***	0.889***			
13- Reflective thinking	0.444***	0.456***	0.422***	0.414***	0.394***	0.311***	0.382***	0.326***	0.342***	0.209***	0.864***	0.873***		
14- PSS mean score	0.525***	0.549***	0.465***	0.502***	0.521***	0.385***	0.427***	0.376***	0.368***	0.327***	0.644***	0.608***	0.530***	

*p < 0.05, **p < 0.01, ***p < 0.001.

A detailed examination of the correlation magnitude indicated that overall academic stress and its eight sub-dimensions were significantly and positively associated with bedtime procrastination. Notably, among the specific stressors, prospect stress exhibited the strongest correlation with bedtime procrastination (*r* = 0.294, *p* < 0.001). This pattern suggests that anxiety regarding future uncertainty constitutes a more critical antecedent of bedtime procrastination behavior than immediate task demands or environmental factors. Conversely, while environmental stress reached statistical significance, it displayed the weakest association with bedtime procrastination relative to the other dimensions.

Furthermore, the potential psychological mechanism variables, specifically the three dimensions of rumination (symptom rumination, brooding, and reflection) and perceived stress, demonstrated moderate-to-high positive correlations with academic stress. In particular, the correlation coefficient between perceived stress and the total academic stress score was 0.525, showing particularly strong associations with the dimensions of competition stress and prospect stress. These findings imply that individuals experiencing elevated academic stress frequently exhibit concurrent high levels of psychological distress and negative cognitive rumination. Concurrently, the results indicated significant positive associations between bedtime procrastination and both perceived stress and the various dimensions of rumination. It is noteworthy that the correlation between perceived stress and bedtime procrastination (*r* = 0.327) was markedly higher than the average correlation coefficients observed between individual academic stress dimensions and procrastination. Statistically, this result provides preliminary evidence suggesting that objective academic burden may not drive procrastination directly; rather, it likely exacerbates bedtime procrastination behaviors indirectly through psychological processes such as individual perceived stress and rumination.

### 3.2 Analysis of demographic differences in bedtime procrastination

This study employed independent samples *t*-tests and one-way analysis of variance (ANOVA) to systematically examine the distributional differences of bedtime procrastination behavior across various demographic variables, with detailed statistical metrics presented in Table 2. The statistical results indicate that demographic characteristics exert a specific and significant influence on bedtime procrastination. Specifically, grade level, physical health status, and the duration of daytime mobile entertainment usage emerged as critical determinants, whereas other characteristics such as gender and place of origin did not demonstrate statistically significant differences.

**TABLE 2:** Differences in bedtime procrastination by demographic characteristics.

Groupings		Mean	SD	F/t	p	η2	*Post-hoc* comparisons
Gender	Male	3.15	0.68	1.191	0.276	0.002	−
	Female	3.21	0.67				
Only child status	Only child	3.23	0.65	1.504	0.22	0.002	−
	Non-only child	3.17	0.68				
Grade	Freshman	3.10	0.66	5.637	< 0.001	0.03	Junior > Freshman, Junior > Sophomore, Junior > Fifth year or above
	Sophomore	3.30	0.68				
	Junior	3.38	0.64				
	Senior	3.24	0.69				
	Fifth year or above	3.00	0.66				
Physical health status	Very good	2.76	0.58	23.855	< 0.001	0.115	Very Good < Good < average/below average/very poor
	Good	3.09	0.58				
	Average	3.37	0.67				
	Below average	3.55	0.80				
	Very poor	3.64	0.78				
Daytime mobile entertainment usage time	Less than 1 h	2.78	0.61	11.783	< 0.001	0.046	Less than 1 h < 1–3 h/3–5 h < more than 5 h
	1–3 h	3.07	0.72				
	3–5 h	3.15	0.59				
	More than 5 h	3.38	0.68				
Major	Humanities and social sciences	3.19	0.63	0.157	0.960	0.001	−
	Science, engineering, and agriculture	3.20	0.71				
	Arts and physical education	3.17	0.63				
	Medicine	3.30	0.45				
	Other	3.15	0.65				
Hometown	Urban	3.21	0.72	0.502	0.681	0.002	−
	County	3.22	0.70				
	Township	3.19	0.60				
	Rural	3.15	0.64				
Relationship status	Single	3.19	0.67	0.477	0.621	0.001	−
	In a relationship	3.17	0.67				
	Married	2.91	0.35				
School tier	Double first class	3.36	0.77	1.098	0.349	0.004	−
	Public university (undergraduate)	3.19	0.67				
	Private/independent	2.93	0.70				
	Vocational/Technical College	3.06	0.15				
Academic performance	Top 25%	3.17	0.66	1.37	0.251	0.006	−
	25–50%	3.15	0.65				
	50–75%	3.23	0.73				
	Bottom 25%	3.34	0.64				

In terms of specific variances, grade level exerted a significant main effect on bedtime procrastination. *Post-hoc* multiple comparisons (LSD) revealed that the severity of bedtime procrastination followed an inverted U-shaped trajectory as grade level increased. Notably, junior students scored significantly higher than both freshmen and students in their fifth year or above, representing the peak level of procrastination across the entire university period. This finding is likely attributable to the dual anxiety stemming from heavy course loads and the approaching pressures of postgraduate entrance exams or employment during the junior year.

Concurrently, physical health status demonstrated the most pronounced inter-group differences (*F* = 23.855,*p* < 0.001), yielding the highest effect size among all observed variables (η^2^ = 0.115). The data exhibited a clear linear gradient in which bedtime procrastination significantly increased as self-rated health declined. Consequently, the procrastination scores of the group reporting “very poor” health were markedly higher than those of the “very good” health group. This suggests that compromised physiological health may deplete individual self-control resources and thereby exacerbate bedtime procrastination.

Furthermore, the duration of daytime mobile entertainment usage served as a significant predictor of bedtime procrastination. The analysis revealed a distinct dose-response relationship wherein extended daytime mobile usage corresponded to increasingly severe bedtime procrastination at night. In particular, the group using mobile phones for over 5 h per day exhibited significantly higher procrastination levels compared to the low-frequency group using phones for < 1 h.

Conversely, variables including gender, only-child status, academic major, place of origin, relationship status, institution level, and academic ranking did not show statistically significant effects on bedtime procrastination. These results suggest that bedtime procrastination behavior possesses a high degree of universality and homogeneity among college students across different genders, family backgrounds, and academic performance levels.

### 3.3 Results of latent profile analysis of academic stress

#### 3.3.1 Model construction and selection

To investigate the heterogeneity of academic stress perception among college students, this study fitted latent profile models ranging from one to five classes using the eight dimensions of academic stress as manifest indicators. The fit indices for each model are detailed in Table 3.

**TABLE 3:** Results of latent profile analysis of academic stress among college students.

Class	AIC	BIC	aBIC	Entropy	LMRTp	BLRTp	Class proportions
1	16937.74	17011.55	16960.75	−	−	−	−
2	13850.20	13965.54	13886.15	0.925	< 0.001	<0.001	0.41/0.59
3	12511.25	12668.11	12560.15	0.933	0.0062	< 0.001	0.93/0.05/0.02
**4**	**11566.60**	**11764.98**	**11628.44**	**0.945**	**0.0001**	**< 0.001**	**0.11/0.32/0.45/0.12**
5	11214.84	11454.73	11289.61	0.939	0.0176	< 0.001	0.11/0.30/0.42/0.14/0.02

Bold values indicate the optimal model selected (the four-profile solution) based on fit indices and theoretical interpretability.

An inspection of the data trends in Table 3 reveals that the information criteria values, specifically AIC, BIC, and aBIC, exhibited a monotonic decline as the number of classes increased. Notably, the magnitude of this decrease attenuated significantly when the number of classes expanded from three to four, demonstrating a clear pattern of diminishing marginal returns. Furthermore, the likelihood ratio tests provided statistical support for the four-profile solution; both the LMRT and BLRT values were < 0.001, indicating that the four-profile model fit the data significantly better than the three-profile model. Additionally, the four-profile model yielded an entropy value of 0.945, the highest among all tested models, which implies an extremely high classification precision. Synthesizing the statistical fit indices with the theoretical interpretability of the latent classes, we selected the four-profile model as the optimal classification solution.

#### 3.3.2 Characterization and naming of latent profiles

**FIGURE 2:**
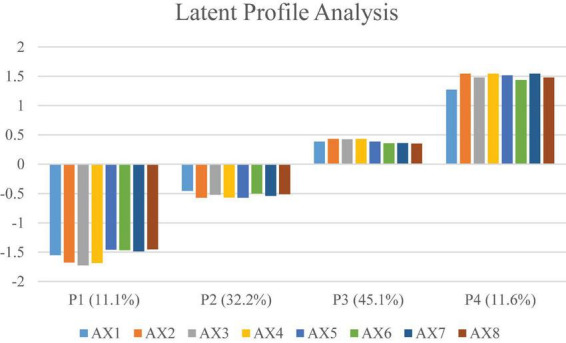
The four academic stress profiles and relative size of the profiles. AX1, Prospect stress; AX2, Goal stress; AX3, Task demand stress; AX4, Competition stress; AX5, Frustration stress; AX6, Parental stress; AX7, Expectation tress; AX8, Environmental stress.

Based on the optimal four-profile model, Figure 2 illustrates the mean score distribution across the eight academic stress dimensions for each latent class. We characterized and named these four subgroups according to their distinct stress patterns.

Profile 1 (P1), comprising 82 participants or 11% of the total sample, is characterized by extremely low scores across all eight dimensions, including prospect stress and competition stress. This pattern suggests that these individuals possess high resilience or low sensitivity to academic stressors; consequently, we designated this cluster as the “Pressure-Insensitive Group.”

Profile 2 (P2) consists of 239 participants, accounting for 32% of the sample. Individuals in this group exhibited scores consistently below the overall mean with a relatively flat distribution. This profile indicates that while these students perceive academic demands, they maintain stress at a low and manageable level, demonstrating good environmental adaptation. Thus, we labeled this cluster the “Well-Adapted Group.”

Profile 3 (P3) represents the largest cluster, comprising 334 participants or 45% of the sample. This group displayed moderate-to-high scores across all dimensions, reflecting that the majority of college students experience a persistent and notable degree of academic burden in their daily lives. Although not yet critical, this stress load is significant; therefore, we named this cluster the “Commonly Burdened Group.”

Profile 4 (P4) includes 86 participants, representing 12% of the sample. This group is distinguished by elevated scores across the entire spectrum of stress dimensions. These individuals concurrently face intense pressure from task demands, expectations, and competition, placing them in a precarious state of psychological resource depletion. Identifying them as a high-risk population requiring priority intervention, we named this cluster the “Overwhelmed Group.”

#### 3.3.3 Verification of discriminant validity

To verify whether the classification of academic stress subtypes holds statistical significance, we conducted an analysis of variance (ANOVA) with the latent class as the independent variable and the scores of the eight academic stress dimensions as dependent variables. As presented in Table 4, the results demonstrate statistically significant differences across all dimensions among the four latent profiles (*p* < 0.001). The extremely high *F*-values observed (e.g., Overall Academic Stress *F* = 1887.724) provide robust empirical evidence confirming the satisfactory discriminant validity of this four-class model.

**TABLE 4:** Analysis of differences in academic stress dimensions across profiles.

Future pressure	Profile	Mean	SD	*F*	*p*
Goal pressure	Stress desensitization	1.7622	0.74577	357.608	<0.001
	Stress adaptation	2.7871	0.66024		
	General load	3.5752	0.53771		
	Stress overload	4.4041	0.44954		
Task demand pressure	Stress desensitization	1.2718	0.36029	918.196	<0.001
	Stress adaptation	2.2917	0.41963		
	General load	3.2096	0.41743		
	Stress overload	4.2392	0.48380		
Competitive pressure	Stress desensitization	1.3360	0.39582	760.830	<0.001
	Stress adaptation	2.3835	0.43793		
	General load	3.1989	0.38899		
	Stress overload	4.1021	0.54625		
Frustration pressure	Stress desensitization	1.2613	0.30101	897.616	<0.001
	Stress adaptation	2.2403	0.39698		
	General load	3.1155	0.40797		
	Stress overload	4.0797	0.48833		
Parental pressure	Stress desensitization	1.1634	0.26875	503.891	<0.001
	Stress adaptation	1.9598	0.44351		
	General load	2.8162	0.55006		
	Stress overload	3.8047	0.68047		
Expectation pressure	Stress desensitization	1.1829	0.36815	396.395	<0.001
	Stress adaptation	2.1095	0.53972		
	General load	2.9251	0.61990		
	Stress overload	3.9302	0.73185		
Environmental pressure	Stress desensitization	1.1789	0.38559	530.718	<0.001
	Stress adaptation	2.0251	0.45969		
	General load	2.8493	0.50249		
	Stress overload	3.9147	0.71251		
Overall academic stress	Stress desensitization	1.1250	0.31732	420.280	<0.001
	Stress adaptation	1.9854	0.45983		
	General load	2.7612	0.58451		
	Stress overload	3.7762	0.76218		
	Stress desensitization	1.3253	0.27659	1887.724	<0.001
	Stress adaptation	2.2815	0.26694		
	General load	3.1201	0.23050		
	Stress overload	4.0766	0.39523		

#### 3.3.4 Predictive effect of academic stress profiles on bedtime procrastination

Having established the classification of academic stress subtypes, we employed the robust BCH method (Bolck, Croon, and Hagenaars’s method) to examine the differential impact of these latent profiles on the distal outcome variable, specifically Bedtime Procrastination (BPS). Table 5 presents the results of the overall Chi-square test and pairwise comparisons.

**TABLE 5:** Relations of the four latent profiles to BPS in the full sample.

Profile	M ± SE	BCH χ ^2^	Overall test
P1 stress desensitization	−0.283 ± 0.132	17.131**	P4 > P3 > P2 > P1
P2 stress adaptation	−0.122 ± 0.066		
P3 general load	0.074 ± 0.051		
P4 stress overload	0.325 ± 0.125		

**p < 0.001.

The analysis revealed a significant association between the different academic stress patterns and bedtime procrastination behavior (Wald χ^2^ = 17.131, *p* < 0.01). Specifically, the pairwise comparisons uncovered a distinct linear gradient: *P*4 > *P*3 > *P*2 > *P*1. This finding indicates that as the characteristics of the profile evolve from “Insensitive” to “Overwhelmed,” the level of bedtime procrastination increases monotonically. Notably, the Overwhelmed Group exhibited the highest standardized level of procrastination (*M* = 0.325), significantly surpassing all other groups; conversely, the Pressure-Insensitive Group demonstrated the lowest level (*M* = −0.283). These results strongly suggest that academic stress constitutes a critical risk factor that exacerbates bedtime procrastination among college students.

### 3.4 Mediation analysis of rumination dimensions

To elucidate the psychological mechanisms underlying the relationship between academic stress subtypes and bedtime procrastination, this study employed the PROCESS macro (Model 4) for SPSS to construct a parallel multiple mediation model. In this model, latent profiles of academic stress served as the independent variables, the three dimensions of rumination functioned as mediating variables, and bedtime procrastination behavior was the dependent variable. Demographic variables previously identified as having significant effects on the dependent variable, such as grade level, physical health status, and daytime mobile phone usage, were strictly controlled. Given the categorical nature of the independent variable, a dummy coding strategy was applied wherein the “Pressure-Insensitive Group” (the lowest stress level) was set as the reference category. The relative effects of the “Well-Adapted Group” (X1), “Commonly Burdened Group” (X2), and “Overwhelmed Group” (X3) were then examined against this baseline.

#### 3.4.1 Regression analysis results

The regression analysis revealed that, relative to the reference group, all three higher-stress groups significantly and positively predicted each dimension of rumination. Specifically, a distinct dose-response relationship was observed where the regression coefficients for symptom rumination, brooding, and reflection increased monotonically with the severity of the stress profile. For instance, the standardized regression coefficient of the Overwhelmed Group on symptom rumination (β = 1.57,*p* < 0.001) was markedly higher than that of the Well-Adapted Group (β = 0.27,*p* < 0.05), indicating that elevated academic stress substantially heightens the risk of engaging in negative cognitive rumination.

Upon introducing the mediating variables, the direct effects of the academic stress profiles on bedtime procrastination ceased to be significant. This suggests that the impact of academic stress on procrastination is primarily transmitted through indirect pathways. It is critical to note that the specific dimensions of rumination exerted divergent influences on the outcome variable. Symptom rumination significantly and positively predicted bedtime procrastination (β = 0.24,*p* = 0.002), acting as a risk factor. Conversely, reflection demonstrated a significant negative predictive effect (β = −0.22,*p* = 0.002), functioning as a protective factor. These findings imply that not all forms of rumination precipitate procrastination; rational reflection may facilitate self-regulation.

#### 3.4.2 Bootstrap verification of mediation effects

The results of the mediation effect test, based on the bias-corrected percentile Bootstrap method (5,000 resamples), are detailed in Table 6. The confidence interval analysis confirmed that, after controlling for relevant demographic covariates, both symptom rumination and reflection played significant mediating roles in the relationship between academic stress subtypes and bedtime procrastination.

**TABLE 6:** Relative mediation effects of symptom rumination and reflection.

Effect	Independent variable	Dependent variable	Estimate	SE	t	
Relative total effect	X1 Well-adapted	BPS	0.12	0.08	1.65	
	X2 Commonly burdened		0.20	0.07	2.72**	
	X3 Overwhelmed		0.31	0.09	3.35***	
Relative direct effect	X1 Well-adapted	BPS	0.09	0.07	1.18	
	X2 Commonly burdened		0.13	0.07	1.70	
	X3 Overwhelmed		0.11	0.10	1.11	
	**Independent variable**	**Dependent variable**	**Estimate**	**SE**	**BootLLCI**	**BootULCI**
Indirect effect (symptom rumination)	X1 Well-adapted	BPS	0.04	0.03	0.002	0.10
	X2 Commonly burdened		0.10	0.04	0.02	0.19
	X3 Overwhelmed		0.26	0.10	0.06	0.46
Indirect effect (reflection)	X1 Well-adapted	BPS	−0.04	0.03	−0.10	−0.0001
	X2 Commonly burdened		−0.09	0.04	−0.17	−0.02
	X3 Overwhelmed		−0.21	0.09	−0.39	−0.05

* Covariates include gender, age, grade, health status, and phone usage; Bootstrap samples = 5,000; **p < 0.01, ***p < 0.001.

Specifically, the indirect effect of symptom rumination was positive, with 95% confidence intervals that did not contain zero (e.g., Overwhelmed Group path: *BootLLCI* = 0.06,*BootULCI* = 0.46). This indicates that academic stress exacerbates bedtime procrastination by triggering symptom rumination. In contrast, the indirect effect of reflection was negative, and its confidence intervals also excluded zero (e.g., Overwhelmed Group path: *BootLLCI* = −0.39,*BootULCI* = −0.05). This negative mediation effect, often interpreted as a suppression effect, suggests that although high academic stress stimulates reflection, this rational, solution-oriented cognitive process acts as a buffer. It partially mitigates the adverse impact of stress on sleep habits, thereby suppressing the occurrence of bedtime procrastination.

### 3.5 Moderated mediation analysis

#### 3.5.1 Testing the moderated mediation model

To further explore the boundary conditions of the mediation mechanism involving rumination, this study introduced “Perceived Stress” (Z) as a moderator. Using Model 59 in the PROCESS macro, which assumes that all paths can be moderated, we examined the moderating role of perceived stress on the trajectories from latent academic stress profiles to rumination and bedtime procrastination. The statistical results are presented in Table 7.

**TABLE 7:** Results of moderated mediation analysis.

	Model 1 (ruminative thinking)			Model 2 (reflective thinking)			Model 3 (sleep procrastination)		
Predictors	*B*	se	*t*	*B*	se	*t*	*B*	se	*t*
Constant	−0.58	0.42	−1.38	−0.56	0.50	−1.13	2.38	0.51	4.69
X1	0.05	0.07	0.72	0.10	0.09	1.13	0.07	0.09	0.73
X2	0.12	0.07	1.64	0.17	0.08	2.02*	0.08	0.09	0.91
X3	0.55	0.10	5.75***	0.64	0.11	5.60***	−0.04	0.12	−0.32
Ruminative thinking							0.18	0.08	2.14*
Reflective thinking							−0.18	0.07	−2.53*
Perceived pressure (Z)	0.49	0.08	5.73***	0.42	0.10	4.18***	0.08	0.11	0.75
X1 × Z	0.02	0.11	0.17	0.04	0.13	0.33	0.05	0.13	0.40
X2 × Z	0.27	0.10	2.63**	0.22	0.12	1.81	0.10	0.13	0.73
X3 × Z	0.26	0.14	1.86	0.04	0.17	0.26	0.38	0.20	1.87
Ruminative thinking × Z							0.09	0.14	0.68
Reflective thinking × Z							−0.06	0.11	−0.55
*R* ^2^	0.50			0.35			0.32		
*F*	37.50			20.02			13.13		
*p*	<0.001			<0.001			<0.001		

*p < 0.05, **p < 0.01, ***p < 0.001.

The model analysis revealed that, after controlling for relevant demographic covariates, perceived stress significantly moderated the first stage of the mediation path, specifically the relationship between the “Commonly Burdened Group” (X2) and symptom rumination (Interaction term *B* = 0.27,*p* = 0.009). This finding implies that for students in the “Commonly Burdened” category, the level of symptom rumination is not static; rather, it is significantly contingent upon the intensity of the individual’s subjective perceived stress.

It is worth noting that the interaction between the “Overwhelmed Group” (X3) and perceived stress was not statistically significant (*p* > 0.05). A plausible explanation for this result is a “ceiling effect.” Since students in the Overwhelmed Group are subjected to extreme objective academic burdens, their symptom rumination may have already reached a saturation point. Consequently, the capacity for subjective perception to buffer or further modulate this high-level rumination is substantially limited.

#### 3.5.2 Simple slope analysis

**FIGURE 3:**
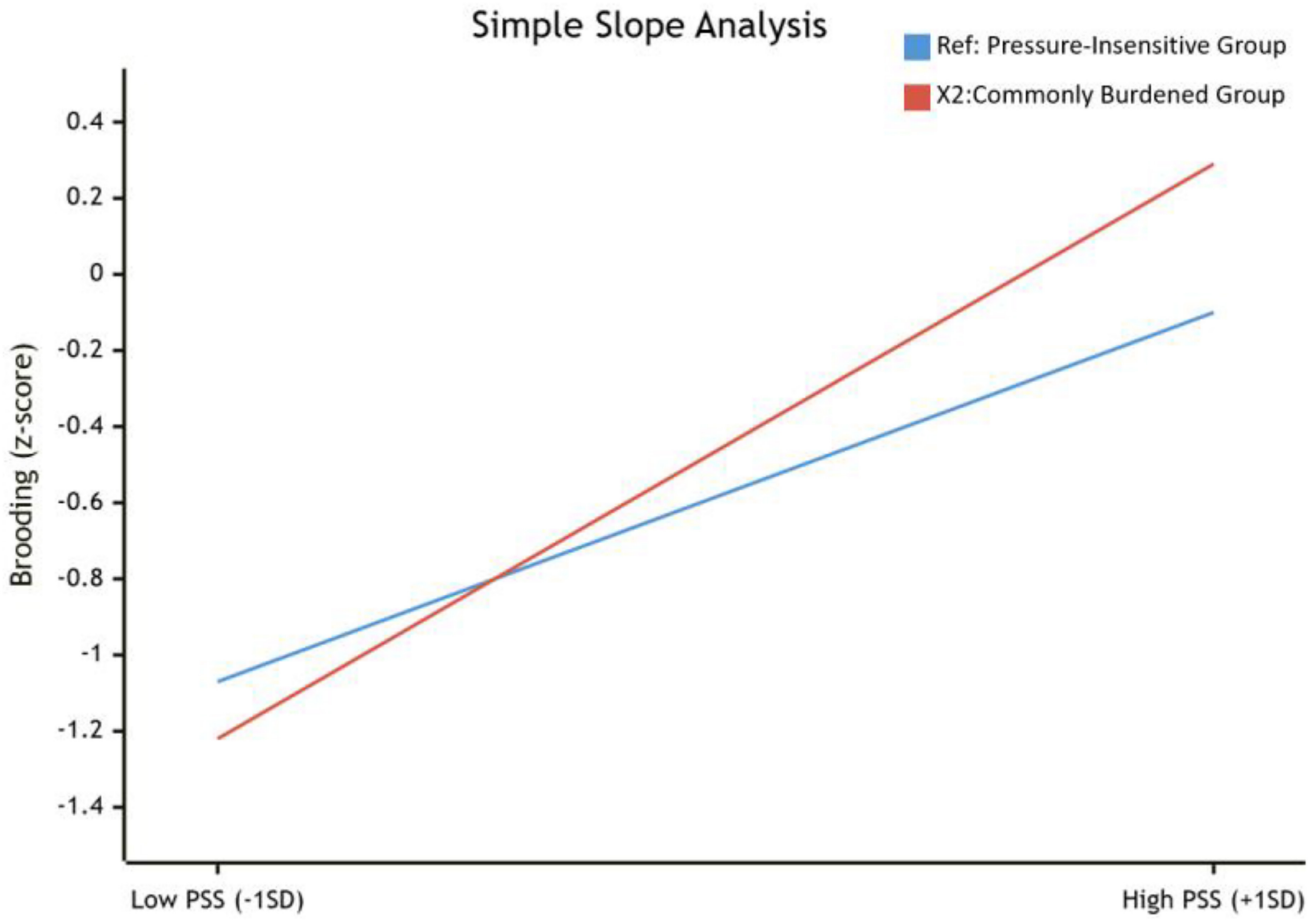
Simple slope plot of the moderating effect.

To decompose the specific pattern of moderation exerted by perceived stress on the relationship between the Commonly Burdened Group (X2) and symptom rumination, a simple slope test was conducted. The results are illustrated in Figure 3.

The slope analysis revealed a distinct “risk activation” pattern. Specifically, at low levels of perceived stress (-1SD), there was no significant difference in symptom rumination levels between the Commonly Burdened Group and the reference group (Pressure-Insensitive Group) (*SimpleSlope* = −0.15,*p* > 0.05). This indicates that when subjective perceived stress remains below a certain threshold, individuals in the “Commonly Burdened” category can maintain psychological equilibrium without exhibiting significant pathological rumination tendencies.

However, at high levels of perceived stress (+1SD), the dynamic shifts fundamentally. Under these conditions, the Commonly Burdened Group exhibited significantly higher levels of symptom rumination compared to the reference group (*SimpleSlope* = 0.39,*p* > 0.01). This finding not only elucidates the positive sign of the interaction term but also profoundly identifies the Commonly Burdened Group as a “stress-susceptible population.” In this context, perceived stress functions as a “risk activator.” As subjective perceived stress escalates, the latent psychological vulnerability of this group is rapidly triggered, resulting in a sharp deterioration in symptom rumination and a cognitive dissonance response that is markedly more severe than that of the insensitive group.

## 4 Discussion

Adopting a person-centered approach, this study unravels the complex mechanisms linking academic stress subtypes to bedtime procrastination behavior among college students. By moving beyond variable-centered assumptions of linearity, this research confirms the population heterogeneity of academic stress and elucidates the opposing mediating roles of different rumination dimensions. Furthermore, it highlights the critical function of perceived stress as a risk activator. This interpretation is also broadly consistent with recent review-level evidence showing that bedtime procrastination is closely linked to psychological distress in university students, particularly perceived stress, highlighting its role as a stress-related self-regulation problem (Azeem et al., 2026; Oyar et al., 2026).

### 4.1 Latent heterogeneity of academic stress and the dominance of prospect anxiety

A primary contribution of this study is the identification of four distinct latent profiles: the Pressure-Insensitive Group, Well-Adapted Group, Commonly Burdened Group, and Overwhelmed Group. This taxonomy empirically supports the theoretical premise that individuals appraise similar objective educational environments through qualitatively distinct psychological lenses (Lu et al., 2017). It is particularly concerning that the Commonly Burdened Group constitutes the largest proportion (45%) of the sample. This distribution suggests that moderate-to-high academic stress has evolved into a normalized ecological feature of contemporary university life, a finding that aligns with recent epidemiological surveys in higher education (Liu et al., 2020).

The analysis further identifies prospect stress as the most potent predictor of bedtime procrastination, surpassing even immediate task demands. This finding holds significant theoretical implications. According to Uncertainty Management Theory, anxiety regarding future outcomes, such as career trajectory and postgraduate entrance exams, consumes more cognitive resources than determinate immediate tasks (Brashers, 2001). In an increasingly competitive society, prospect anxiety acts as a chronic stressor that cannot be resolved through immediate action. Consequently, individuals resort to procrastination as a form of emotion-focused coping to temporarily suspend their worries (Xu et al., 2021). This corroborates the conceptualization by Sirois of procrastination as a mechanism for short-term mood repair at the expense of long-term wellbeing (Sirois and Pychyl, 2013).

Additionally, the Overwhelmed Group exhibited uniformly high scores across all dimensions. This pattern indicates a state of systemic psychological saturation. These students are not merely struggling with a single stressor but are experiencing a cumulative allostatic load, rendering them the most vulnerable population requiring urgent intervention (Asif et al., 2020).

### 4.2 Cascading effects of stress on sleep: a conservation of resources perspective

The results demonstrated a distinct linear gradient wherein bedtime procrastination intensified as the profile phenotype evolved from the Insensitive profile to the Overwhelmed profile. This phenomenon can be robustly interpreted through the Conservation of Resources (COR) Theory (Hobfoll, 1989). According to COR principles, individuals in high-stress profiles must invest substantial cognitive and emotional resources to cope with academic demands during the day. This compensatory effort leads to a state of ego depletion by nighttime (Baumeister et al., 2007). Consequently, bedtime procrastination emerges not merely as poor time management but as a form of revenge behavior, representing a desperate attempt to reclaim personal autonomy and leisure time after a day of resource exhaustion (Kroese et al., 2014).

Demographic analyses further nuanced this picture by revealing an inverted U-shaped trajectory for procrastination across grade levels, peaking in the junior year. This “junior crisis” likely stems from the dual pressures of intense coursework and critical career decision-making, which represent a pinnacle of resource drain (Kadison and DiGeronimo, 2004). Similarly, the finding that students with poorer physical health exhibit higher procrastination supports the hypothesis of mind-body resource pooling. Compromised physiological health diminishes the volition required to resist the temptation of digital entertainment at night (Exelmans and Van den Bulck, 2021b). Collectively, these findings imply that addressing bedtime procrastination requires holistic interventions that restore both physiological and psychological capital.

### 4.3 The double-edged sword of rumination: maladaptive fixation vs. adaptive reflection

One of the most compelling findings of this study is the divergence in the mediating roles of rumination dimensions. Specifically, symptom rumination functioned as a risk factor, acting as a positive mediator, whereas reflection functioned as a protective buffer, acting as a negative mediator. These findings suggest that the cognitive pathways linking academic stress to bedtime procrastination are not uniform, but instead depend on the specific form that rumination takes.

On one hand, academic stress exacerbated procrastination by triggering symptom rumination. This aligns with the Response Styles Theory proposed by Nolen-Hoeksema (1991). Students under high pressure often fall into a cycle of passive and repetitive dwelling on their distress. This maladaptive cognitive pattern significantly heightens pre-sleep arousal, making sleep onset difficult and prompting the individual to engage in mobile phone use as a distraction technique (Nota and Coles, 2015; Rogers and Barber, 2019).

In addition, the role of brooding also deserves attention. Conceptually, brooding represents a maladaptive and self-critical form of repetitive thinking characterized by passive comparison between one’s current situation and unachieved standards (Takano and Tanno, 2009; Whitmer and Gotlib, 2013). Such thinking may intensify negative self-evaluation, sustain emotional fixation, and further undermine effective emotion regulation before sleep. Although brooding did not emerge as a significant mediator in the final model, its theoretical relevance should not be overlooked. One possible explanation is that, when symptom rumination and reflection were considered simultaneously, the unique effect of brooding may have been partially absorbed by other rumination-related cognitive components. Therefore, brooding may still represent an important maladaptive cognitive tendency in the context of academic stress, even if its independent indirect effect was not statistically significant in the present study.

On the other hand, reflection demonstrated an unexpected suppression effect. Unlike symptom rumination and brooding, reflection involves active cognitive restructuring and purpose-driven self-inquiry (Treynor et al., 2003). The data suggest that while high stress does trigger reflection, this rational processing facilitates problem-solving and emotional regulation. Such adaptive processing subsequently reduces the urge to procrastinate (Burwell and Shirk, 2007). This helps explain why the Commonly Burdened Group, despite facing significant pressure, did not uniformly exhibit extreme procrastination, as a subset of these individuals likely engaged adaptive reflective mechanisms. Taken together, these findings challenge the monolithic view of rumination and underscore the importance of distinguishing among symptom rumination, brooding, and reflection. From a practical perspective, interventions targeting bedtime procrastination should not only reduce maladaptive fixation on distress and self-critical brooding, but also foster more adaptive reflective processing, so as to improve emotional regulation and sleep-related self-control.

### 4.4 Perceived stress as a risk activator: the susceptibility of the commonly burdened

The moderated mediation analysis provided critical insights into the boundary conditions of these effects. Perceived stress significantly moderated the path from the *Commonly Burdened Group* to symptom rumination, revealing a Risk Activation Model (Luthar et al., 2000).

For students in the Commonly Burdened Group, objective academic loads exist but are not inherently catastrophic. In this context, subjective Perceived Stress acts as a decisive gating mechanism (Cohen et al., 1983). When perceived stress is low, these students maintain psychological equilibrium, supporting the buffering hypothesis of resilience (Rutter, 1987). However, once subjective perceived stress escalates, their latent psychological susceptibility is rapidly activated. This activation causes symptom rumination to spike to levels comparable to the Overwhelmed Group (Hammen, 2005).

Intriguingly, this interaction was not significant for the *Overwhelmed Group*. This null finding is likely attributable to a Ceiling Effect (Wang et al., 2011). For individuals chronically exposed to extreme objective demands, psychological defense systems may be overwhelmed regardless of subjective appraisal. The objective environment exerts such a potent influence that it bypasses the regulatory function of subjective perception, directly driving high levels of rumination and procrastination (Varma and Knight, 2021). This distinction has profound practical utility. It suggests that cognitive reappraisal interventions may be highly effective for the majority of students who fall into the Commonly Burdened category. Conversely, the minority in the Overwhelmed category requires structural changes to reduce objective load, such as academic support systems, to break the vicious cycle (Ribeiro et al., 2018).

### 4.5 Limitations and future directions

While this study offers valuable insights, several limitations warrant consideration. First, the cross-sectional design precludes causal inference. Future research should employ diary studies or longitudinal designs to capture the immediate and within-person effects of daily academic stress on subsequent bedtime procrastination (Ohly et al., 2010). Second, because all variables were measured using self-report questionnaires, common method bias cannot be entirely ruled out. Although the present study did not include a separate *post-hoc* statistical test specifically targeting common method bias, the observed findings showed differentiated and theory-consistent patterns rather than uniformly inflated same-source associations (Schnauber-Stockmann and Naab, 2019). For example, the latent profile analysis identified distinct academic stress subtypes, the BCH results revealed graded differences in bedtime procrastination across profiles, symptom rumination and reflection showed opposite effects in the mediation model, and only a specific interaction path was significant in the moderated mediation model. These patterns suggest that the main findings are unlikely to be fully attributable to a single common method factor. Future studies are encouraged to adopt multi-wave, multi-source, or longitudinal designs to further reduce this concern. Finally, future intervention studies could leverage the classification system developed here to design stratified interventions that specifically target the transformation of maladaptive symptom rumination into adaptive reflection (Watkins, 2016).

## 5 Conclusion

By integrating a person-centered latent profile approach with a variable-centered mechanism analysis, this study provides a comprehensive reconstruction of the relationship between academic stress and bedtime procrastination among college students. The results move beyond simple linear associations to reveal the nuanced ways in which objective burdens and subjective cognitive processes jointly precipitate sleep self-regulation failures.

First, the identification of four distinct latent profiles reveals significant heterogeneity in how students perceive academic demands. The findings highlight that the Overwhelmed Group faces a systemic collapse of coping resources, whereas the Commonly Burdened Group represents the normalized state of moderate pressure in higher education. Furthermore, the dominance of prospect stress as a predictor underscores that anxiety regarding future uncertainty, rather than immediate task load, constitutes the primary cognitive burden driving sleep delay. This supports the interpretation of bedtime procrastination as a compensatory mechanism for resource depletion caused by future-oriented anxiety.

Second, the study elucidates the dual nature of rumination within the stress-procrastination relationship. The results demonstrate that different dimensions of rumination exert opposing effects. Symptom rumination functions as a maladaptive accelerator that exacerbates procrastination by depleting cognitive resources. In contrast, reflection serves as a protective cognitive buffer that mitigates the adverse effects of stress through adaptive problem-solving. This distinction challenges the traditional view of rumination as a monolithic negative construct and suggests that fostering reflective thinking can be a vital component of resilience.

Finally, the moderated mediation analysis identifies perceived stress as a critical risk activator. For the majority of students falling into the Commonly Burdened Group, subjective stress appraisal acts as a gating mechanism that determines whether objective pressure transforms into pathological procrastination. Conversely, the absence of this moderation effect in the Overwhelmed Group suggests a ceiling effect where objective stressors overpower subjective regulation.

Collectively, these findings support a stratified intervention framework. Effective countermeasures must move beyond generic time management advice to address the specific resource deficits of different profiles. For the Overwhelmed Group, structural changes to reduce academic load are imperative. For the Commonly Burdened Group, cognitive interventions aimed at reducing symptom rumination and enhancing adaptive reflection are likely to yield the best outcomes. Ultimately, resolving bedtime procrastination requires a dual focus on alleviating future-oriented anxiety and fostering adaptive reflective thinking to restore the psychological resources necessary for healthy sleep regulation.

## Data Availability

The original contributions presented in the study are included in the article/supplementary material, further inquiries can be directed to the corresponding author.
